# O033. TDCs treatment: long term results in drug resistant migrainous patients

**DOI:** 10.1186/1129-2377-16-S1-A178

**Published:** 2015-09-28

**Authors:** Giorgio Dalla Volta, Daniela Carli, Paola Zavarise, Fabio Antonaci

**Affiliations:** Brescia Headache Center, Istituto Clinico Città di Brescia, Brescia, Italy; Headache Centre, C. Mondino National Institute of Neurology Foundation, IRCCS, Deparment of Brain and Behavioral Sciences University of Pavia, Pavia, Italy

## Introduction

Transcranial Direct Current Stimulation (tDCS), a non invasive, neuroplasticity-generating brain stimulation tool, is increasingly used for therapeutic purposes in neurological and psychiatric diseases with pathological alterations of cortical excitability and activity. A multicenter observational study using tDCS in the treatment of migraine was carried out.

## Materials and methods

As part of a larger study, 60 patients with chronic migraine, recruited from the Headache Center of the Istituto Clinico Città di Brescia, and Pavia Headache Center Patients, underwent electrical (n=30) or sham (n=30) stimulation, in 5 treatment sessions every other day for 10 minutes and at follow-up at t30, t60, t90 and t120. After 6 months from the last stimulation we selected all patients who had achieved and maintained a benefit from the treatment (n=12). They underwent electrical stimulation in 5 treatment sessions and at follow-up (t30 and t90).

For each patient, headache frequency, duration and intensity (VAS), headache days per month and the response to symptomatic therapy had also been evaluated; besides, a list of the 5 main precipitating factors was collected.

## Results

Primary endpoint was the reduction of at least 50% of the headaches’ clinical parameters (frequency, severity, use of medication). Secondary endpoint was the assessment of plausible decreasing threshold of cortical excitability by evaluating response to trigger factors.

At t30 a reduction of at least 50% of the parameters evaluated in almost 75% of the patients was observed, while at t60 the benefit was maintained for approximately 40% of treated patients, as well as at t90 and t120.

Three months after the last stimulation, headache frequency, headache days, duration and intensity (VAS), per month were evaluated for each patient: 80% of the patients (n=9) obtained a further reduction of at least 50% of the headache parameters evaluated.

## Discussion

There is increasing evidence that brainstem, as well as cortical dysfunction, are basically involved in the complex pathophysiology of migraine. Modified neuronal excitability might be one possible explanation of the efficacy of both pharmacological treatment and tDCS treatment.

## Conclusions

tDCS has been introduced as a non invasive tool to guide neuroplasticity and to modulate cortical function by tonic stimulation with weak direct currents. Studies involving patients affected by chronic pain confirm that tDCS can produce long-lasting pain relief.

Written informed consent to publish was obtained from the patient(s).

